# An overview of protein *N*-glycosylation diversity in microalgae

**DOI:** 10.3389/fpls.2025.1669918

**Published:** 2025-09-25

**Authors:** Julia van Bockstaele-Fuentes, Narimane Mati-Baouche, Josselin Lupette, Nesrine Gargouch, Elodie Rivet, Patrice Lerouge, Muriel Bardor

**Affiliations:** ^1^ Université de Rouen Normandie (UNIROUEN), Normandie Univ, GlycoMEV UR 4358, SFR Normandie Végétal FED 4277, Innovation Chimie Carnot, IRIB, GDR CNRS Chemobiologie, Rouen, France; ^2^ ALGA BIOLOGICS, CURIB, Mont Saint Aignan Cedex, France

**Keywords:** N-glycosylation, protein, glycosyltransferase, microalgae, N-glycan pathway

## Abstract

Microalgae are unicellular photosynthetic eukaryotic organisms that arose from distinct endosymbiotic events yielding a wide phylogenetic diversity. In contrast, a single lineage of green algae charophytes gives rise to all land plants. A large number of *N*-glycan structures were already characterized but the study of glycans *N*-linked to proteins in these unicellular organisms only recently begun and concerns a limited number of species. These structures differ to a large extent from known structures and exhibit various glycan decorations not reported so far in land plants. However, many pieces of the puzzle are still missing for a full understanding of the protein *N*-glycosylation biosynthesis in microalgae despite the structural elucidation of lipid-linked oligosaccharide precursors and the biochemical characterization of some Golgi glycosyltransferases. In the present review, we will give an overview of the recently published data on protein *N*-glycosylation in microalgae which enable to draw an updated picture of this sophisticated cellular process leading to a huge diversity of *N*-glycan structures. In this work, we will also highlight the arabinosylation and xylosylation of *N*-glycans in plants and microalgae.

## 1 Introduction

Microalgae are unicellular eukaryotic organisms, mainly photosynthetic, that live in a wide range of habitats where water and light are available. Microalgae gathers over 200,000 species classified in various different phyla (Guiry, 2024). Chlorophytes, glaucophytes and rhodophytes are the products of an endosymbiotic event in which an eukaryotic cell has engulfed a photosynthetic cyanobacterium (De Clerck et al., 2012; Thoré et al., 2023). Then, a secondary endosymbiosis led in one end to euglenophytes and in another end to cryptophytes, haptophytes and stramenopiles (heterokontophytes), including diatoms (Archibald, 2012). For some of these phyla, a third endosymbiosis event occurred (Yoon et al., 2005; Thoré et al., 2023). These successive endosymbiosis events have led to a wide phylogenetic diversity among microalgae phyla. In contrast, a single lineage of the green algae charophyte gave rise to all land plants (Delwiche and Cooper, 2015). Among fundamental biological processes occurring in microalgae, the investigation of glycans *N-*linked to proteins recently received a particular attention from the scientific community. To date, several published articles allow to depict a first picture of *N*-glycan structures and have provided insights on the *N*-glycosylation pathways of proteins in microalgae, enabling a comparative analysis of these data with those from other eukaryotes from an evolutionary point of view (Toustou et al., 2022).

Beyond basic research on the protein *N*-glycosylation processing occurring in these unicellular organisms, expression of therapeutic proteins, namely biologics, in microalgae received recently an increasing interest for biotechnological applications. Together with the implementation of microalgae culture platforms dedicated to the production of high-value molecules, several studies have demonstrated that these unicellular eukaryotic organisms are able to efficiently express functional biologics (Banerjee and Ward, 2022). For instance, the production of functional full-length monoclonal antibodies was achieved in the diatom *Phaeodactylum tricornutum*, thus demonstrating that these unicellular eukaryotes are able to synthesize and assemble complex proteins in their secretory system (Hempel et al., 2011, 2012, 2017; Vanier et al., 2015). Moreover, microalgae benefit from their specific advantages, such as a high growth rate, easy cultivation in photobioreactors and low production costs. However, the production of biologics in microalgae must face up the concern of their *in vivo* biological efficacy and human-compatibility. Indeed, regardless the expression system used as a factory, the *N*-glycosylation of the recombinant therapeutic protein is a major issue because appropriate *N*-glycosylation of the protein must be performed to assure its folding and function. Moreover, non-human glycoepitopes introduced by the expression system on *N*-glycans of biologics may induce immune responses in a therapeutic context (Bardor et al., 2003). To overcome such challenges, efficient glycoengineering strategies were carried out in plants either to erase differences between plant and human *N-*glycan structures or to introduce missing glycoepitopes required for the bioactivity of the plant-derived biologics (Strasser, 2023). This glycoengineering was performed by knock-out strategies through the inactivation of plant Golgi glycosyltransferases (GT) responsible for the transfer of immunogenic glycoepitopes on glycans and/or the complementation of plants with Golgi enzyme sequences (knock-in strategy) able to introduce the missing mammalian glycoepitopes on protein *N*-glycans (Strasser, 2023). The production of biologics in microalgae will also likely require the implementation of engineering strategies as performed in plants to enable the production of microalgae-derived therapeutic proteins carrying human-compatible *N-*glycans. This first requires an in-depth inventory and understanding of protein *N-*glycosylation pathways in microalgae, a prerequisite before any implementation of appropriate glyco-engineering strategies.

In the present review article, we will draw up an overview of recent published data on the protein *N-*glycosylation in microalgae. Then, we will point out on the arabinosylation and xylosylation of *N-*glycans, a structural feature common to plants and microalgae.

## 2 Overview on plant and microalgae protein *N*-glycosylation

**Figure 1:**
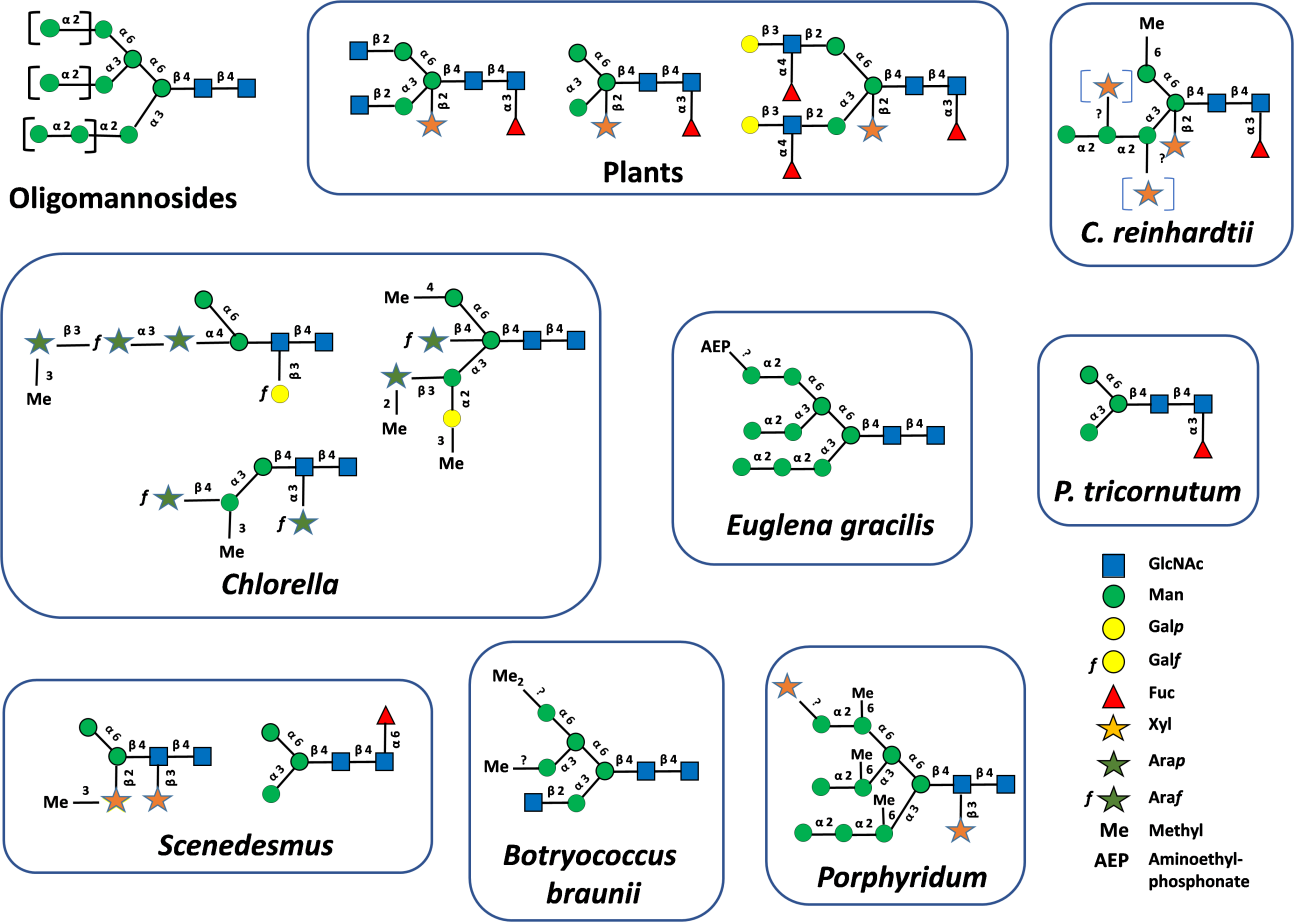
Oligomannosides found *N*-linked to plant and microalgae glycoproteins. Mature protein *N*-linked glycans found in plants and microalgae (Levy-Ontman et al., 2011; Baïet et al., 2011; Mathieu-Rivet et al., 2013; O’Neill et al., 2017; Schulze et al., 2017; Lucas et al., 2020; Mócsai et al., 2020a, 2020b, 2021, 2025). Structures were drawn according to Varki et al. (2015). GlcNAc, *N*-acetylglucosamine; Man, mannose; Gal*p*, galactopyranose; Gal*f*, galactofuranose; Fuc, fucose; Xyl, xylose; Ara*p*, arabinopyranose; Ara*f*, arabinofuranose; Me, methyl; AEP, AminoEthylPhosphonate.

In plants, oligomannosides (also named high-mannose-type glycans) *N-*linked to proteins are composed of a chitobiose unit composed of two *N*-acetylglucosamine (GlcNAc) units linked by a β(1→4) glycosidic bond (GlcNAcβ(1→4)GlcNAc) attached to the Asn residue of proteins. Five to nine mannose (Man) residues are linked to this disaccharide to yield Man_5_GlcNAc_2_ to Man_9_GlcNAc_2_ (**Figure 1**). In addition, mature *N*-glycans resulting from the Golgi processing of oligomannosides are composed of a core GlcNAc_2_Man_3_GlcNAc_2_ substituted by β(1,2)-xylose (Xyl) and/or α(1,3)-fucose (Fuc) epitopes. In addition, glycans lacking the two terminal GlcNAc residues or having one or two Lewis^a^ ((Fucα1→4)Galβ1→3GlcNAc) extensions are found on plant *N-*glycoproteins (**Figure 1**). Whilst the investigation of protein *N-*linked glycans in microalgae only began recently and concerned a restricted number of microalgae species, a wide variety of protein *N-*linked glycans were already characterized. These investigations demonstrated that *N-*glycan structures attached to the proteins of microalgae largely diverge from the one of plants (**Figure 1**). However, despite their structural differences, all microalgae *N*-glycans are composed of at least a Man_2_GlcNAc_2_ or Man_3_GlcNAc_2_ motif ((Manα1→6)(Manα1→3)Manβ1→4GlcNAcβ1→4GlcNAcβ1→) linked to asparagine (Asn) residues of proteins indicating that their biosynthesis shares a common origin with plant and other eukaryotic *N-*glycosylation pathways (**Figure 1**). In addition to oligomannosides, oligosaccharides *N-*linked to proteins of microalgae are composed of Man_2_GlcNAc_2_ to Man_9_GlcNAc_2_ that are decorated with various pentoses, such as Xyl, arabinopyranose (Ara*p*) and arabinofuranose (Ara*f*)), hexoses such as galactopyranose (Gal*p*) and galactofuranose (Gal*f*), Fuc, *O*-methyl (Me) or *O*-aminoethylphosphonate (AEP) groups located at various positions (Levy-Ontman et al., 2011; Baïet et al., 2011; Mathieu-Rivet et al., 2013; O’Neill et al., 2017; Schulze et al., 2017; Lucas et al., 2020; Mócsai et al., 2020a, 2020b, 2021, 2024) (**Figure 1**). As an illustration, Xyl residues were identified located on the β-mannose and/or on the penultimate GlcNAc residue of the chitobiose, as well as on outer Man residues (**Figure 1**). Moreover, it is worth noting that some mature *N*-glycans are structurally based on a non-canonical Man_5_GlcNAc_2_ having a linear trimannosyl sequence on the α(1,3)-mannose arm, as exemplified by *Chlamydomonas* mature *N*-glycans represented in **Figure 1**. In contrast to the *N*-glycosylation process that is highly conserved in plants (Wilson et al., 2001), this wide diversity likely results from the divergent evolution between microalgae phyla as postulated for other cellular processes (Thoré et al., 2023).

The study of the cellular organization in microalgae demonstrated that they possess a secretory system with well-defined ER and Golgi apparatus (Domozych, 1991; Giddings, 2003; Galas et al., 2021). Moreover, some GTs of the *N*-linked glycan processing were localized in specific sub-compartments of the Golgi apparatus suggesting a compartmentation of glyco-enzymes as reported for other eukaryotes (Zhang et al., 2019). As microalgae *N*-glycans are based on an Asn-linked Man_3_GlcNAc_2_ motif and according to the dogma on protein *N-*glycosylation, we thus postulate that the *N*-glycosylation process occurs in microalgae along this secretory pathway with the biosynthesis and transfer in the endoplasmic reticulum (ER) of an oligomannosidic precursor on proteins *via* a well-conserved process that plays a pivotal role for protein folding and quality control. Properly folded glycoproteins are then allowed to continue their transit to the Golgi apparatus where first occurs the trimming of Man residues and then the processing into mature *N-*glycans by a set of specific Golgi glycoside hydrolases (GH) and GTs that enable the protein to acquire organism-specific functions.

## 3 Plant and microalgae *N*-glycosylation pathways

The *N-*glycosylation of proteins in plant has been investigated intensively since the early eighties. GH and GT sequences, their localization within the secretory system and their substrate specificity are now well-documented (Strasser, 2016). With regards to microalgae, protein *N-*glycosylation was only recently investigated with pioneer papers published around 2010. To date, our understanding of this essential cellular processing is mainly based on a few mature *N-*glycans and two lipid-linked oligosaccharides that were structurally identified in the genera *Chlamydomonas, Phaeodactylum, Chlorella* and *Porphyridium*. Cellular localization of enzymes involved in the protein *N-*glycosylation pathway in microalgae and their substrate specificity are currently poorly documented and are still a matter of debate. We will summarize in this chapter what was demonstrated or postulated on the protein *N-*linked glycosylation in microalgae in comparison to plants, starting from the ER steps to the final decoration of mature *N*-glycans in the Golgi apparatus.

### 3.1 *N*-linked glycosylation of proteins in the ER

**Figure 2:**
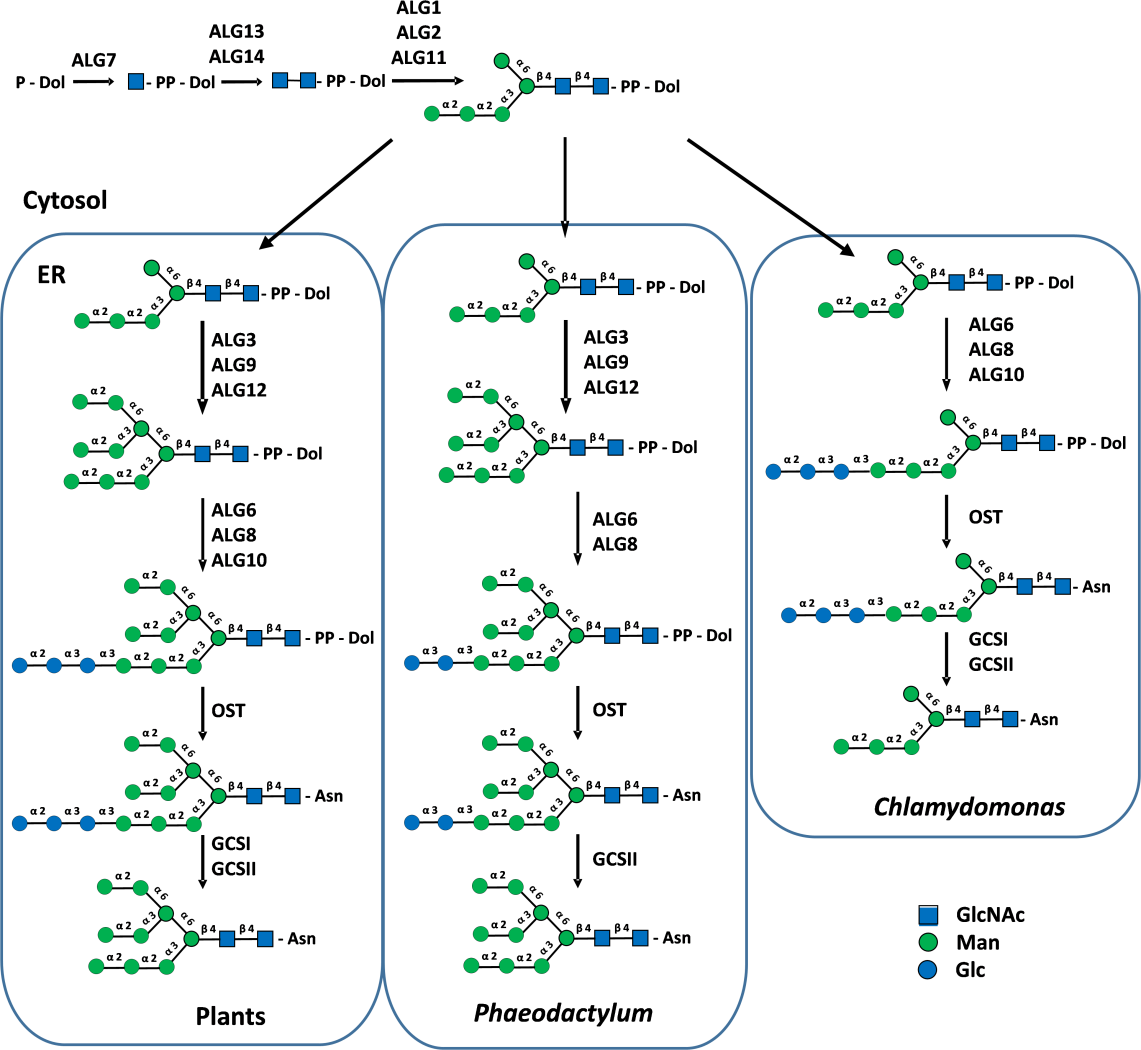
Cytosolic and ER biosynthesis of protein *N*-linked glycans in plants, *P. tricornutum* and *C. reinhardtii*. PP-Dol, dolichol pyrophosphate. Asn, asparagine residue of *N*-glycosylation sites. ALG, Asparagine-Linked Glycosylation; OST, oligosaccharyltransferase complex; GCSI and II, α-glucosidases I and II. Precursor lipid-linked oligosaccharides were biochemically characterized in *P. tricornutum* and *C. reinhardtii* (Lucas et al., 2018). Structures were drawn according to Varki et al. (2015).

In plants, protein *N*-glycosylation occurs along the secretory pathway and involves ER and Golgi biosynthetic events. ER *N*-glycosylation steps consist in the biosynthesis of an oligosaccharide precursor on a dolichol pyrophosphate (PP-Dol) anchored in the ER membrane and then in its transfer onto the asparagine residues of Asn-X-Ser/Thr/Cys *N*-glycosylation consensus sites, although the consensus site Asn/X/Cys is rather rare. In this sophisticated biological process, Man_5_GlcNAc_2_-PP-Dol is first stepwise synthesized on the cytoplasmic face of the ER. After translocation of Man_5_GlcNAc_2_-PP-Dol by a flippase from the cytoplasmic to the luminal face of the ER, the completion of the synthesis of Glc_3_Man_9_GlcNAc_2_-PP-Dol takes then place by action of ER-resident mannosyl- and glucosyltransferases (**Figure 2**). This two-step biosynthesis involves a set of cytosolic or ER enzymes called Asparagine-Linked Glycosylation (ALG) (**Figure 2**). Glc_3_Man_9_GlcNAc_2_ of the lipid-linked oligosaccharide is then transferred *en bloc* by the oligosaccharyltransferase complex (OST) onto the amide group of Asn residues of *N*-glycosylation consensus sites. Then, the trimming of terminal glucose (Glc) residues into Man_9_GlcNAc_2_ by α-glucosidases GCSI and GCSII (**Figure 2**), together with the interactions between the glycoprotein and ER-resident chaperones ensure its folding and quality control before transport into the Golgi apparatus (Strasser, 2016).

**Figure 3:**
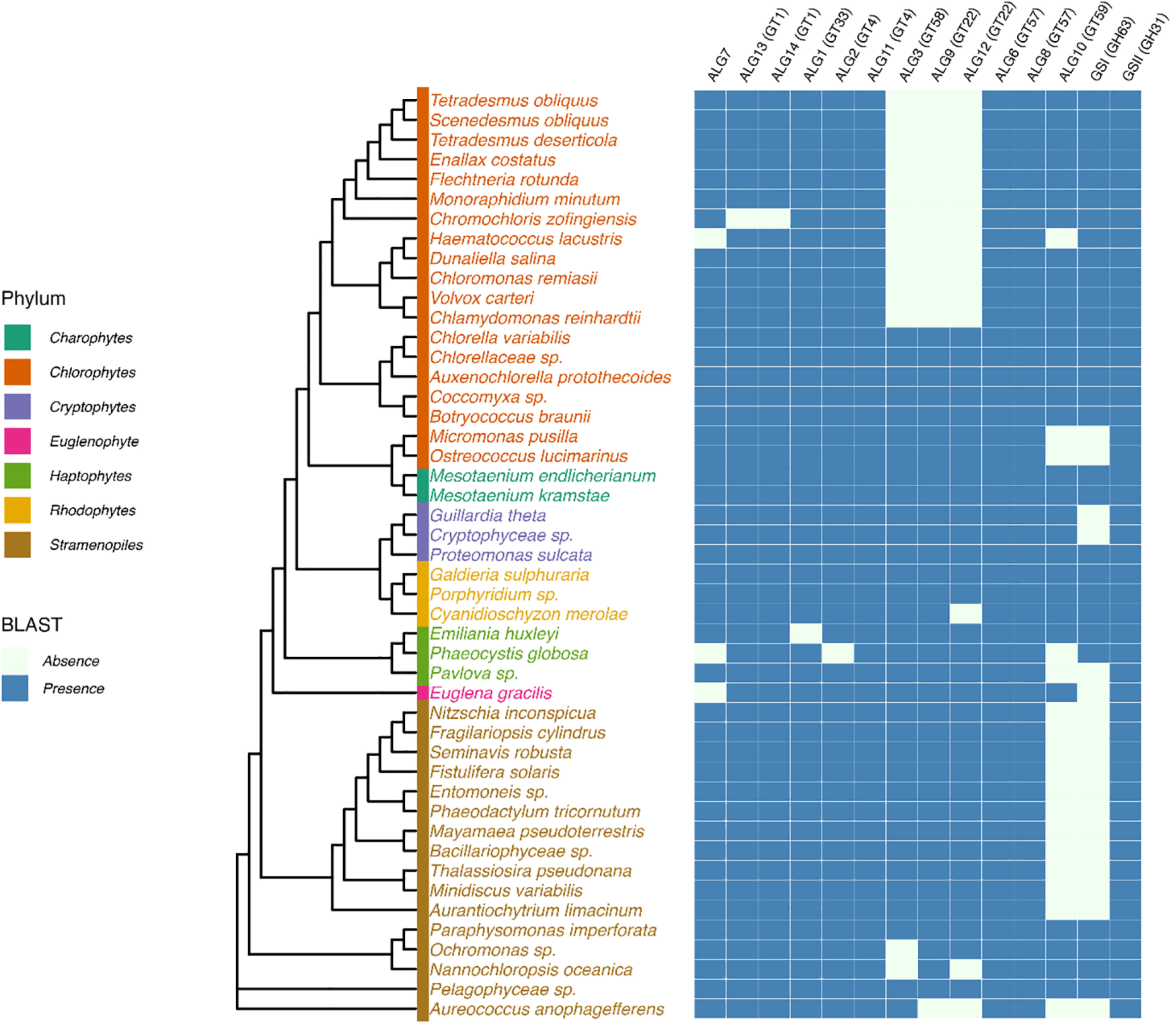
Prediction of putative cytosolic and ER enzymes of the *N-*glycosylation pathway in genomes of microalgae. Protein sequences were identified by BLASTp search in microalgae genomes using genes encoding Arabidopsis glyco-enzymes as query sequences. UniProt assession numbers of proteins used as query sequences: *Arabidopsis thaliana*: ALG1, Q8L7M0; ALG2, F4IBV4; ALG3, O82244; ALG6, Q9FF17; ALG8, O80505; ALG9, Q9FZ49; ALG10, Q8L638; ALG11, Q9XEE9; ALG12, A8MR93; ALG13, O23514; ALG14, Q84R09; GCSI, F4HTM3; GCSII, Q84M89. Cladogram was drawn based on 18S rDNA sequence homology (Branch lengths are non-informative). Sequences were retrieved from the PR2 database (Guillou et al., 2013). Multiple sequence alignment was performed using MAFFT with default parameters (Katoh et al., 2002) and the phylogenetic tree was inferred using maximum likelihood in IQ-TREE 2 (Minh et al., 2020). The tree was visualized and annotated with the R package ggtree (Yu et al., 2017).

Most genes encoding proteins involved in the biosynthesis of the lipid-linked oligosaccharide, its transfer to the protein and the quality control of newly synthesized glycoproteins are predicted in genomes of microalgae (Baïet et al., 2011; Levy-Ontman et al., 2014; Liu et al., 2021; Behnke et al., 2021; Dehghani et al., 2024). In the context of this paper, we also investigated 40 newly available genomes from different phyla to enlarge the overview of putative ALGs and glucosidases GCS I and II in microalgae and to determine whether microalgae within a given phylum share common enzyme repertoires (**Figure 3**). In addition, lipid-linked oligosaccharides have been biochemically characterized in the diatom *Phaeodactylum tricornutum* and the green microalgae *Chlamydomonas reinhardtii* (Lucas et al., 2018). These studies demonstrated that the lack of some ALG gives rise to truncated *N*-glycan precursors in microalgae in comparison with the plant lipid-linked oligosaccharide. For instance, a precursor Glc_2_Man_9_GlcNAc_2_-PP-Dol lacking the outer α(1,2)-linked Glc residue of the triglucosyl sequence was identified in *P. tricornutum* after its isolation from the microsomal fraction and analysis by mass spectrometry (Lucas et al., 2018). This lipid-linked oligosaccharide structure is in accordance with the lack of the predicted glucosyltransferase ALG10 and ER-resident α-glucosidase I (GCS I) in this diatom. These two enzymes are respectively responsible for the transfer of the terminal α(1,2)-linked Glc on the precursor oligosaccharide and then its removal during the quality control process of glycoproteins (Baïet et al., 2011) (**Figures 2** and **3**). On the basis of bio-informatics analyses, same conclusions can be drawn for other diatoms (**Figure 3**). This suggests that in diatoms the ER quality control slightly differs from the one reported in plants but yields the same Man_9_GlcNAc_2_ *N*-linked to proteins leaving the ER (**Figure 2**).

**Figure 4:**
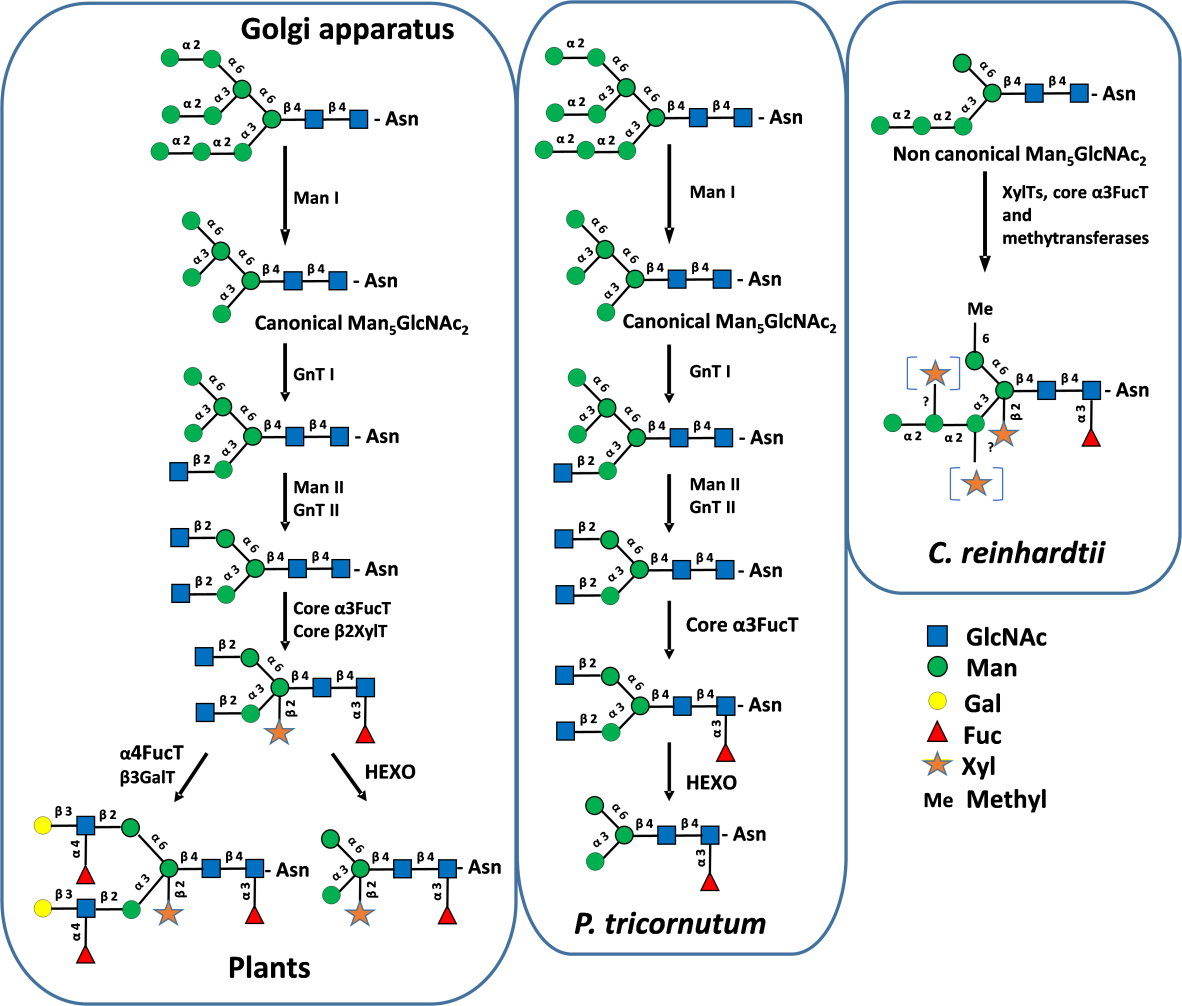
Protein *N*-glycosylation pathways in the Golgi apparatus in plants, *P. tricornutum* and *C. reinhardtii.* GnT I *and II, N*-acetylglucosaminyltransferases I and II; Man I and II, α-mannosidases I and II; FucT, fucosyltransferase; XylT, xylosyltransferase; GalT, galactosyltransferase; HEXO, *N*-acetylhexoaminidase. GlcNAc, *N*-acetylglucosamine; Man, mannose; Gal, galactose; Fuc, fucose; Xyl, xylose; Me, methyl. Mature *N-*glycans were biochemically characterized in *P. tricornutum* and *C. reinhardtii* (Baïet et al., 2011; Mathieu-Rivet et al., 2013; Lucas et al., 2018, 2020). Structures were drawn according to Varki et al. (2015).

*C. reinhardtii* lacks ALG3, ALG9 and ALG12 and this results in the biosynthesis in this green microalgae of a Glc_3_Man_5_GlcNAc_2_-PP-Dol missing the four Man residues located on the α(1,6)-mannose arm of the core *N-*glycan (Lucas et al., 2018) (**Figure 2**). Then, the linear trimannosyl sequence (Manα1→2Manα1→2Manα1→) of this truncated lipid-linked oligosaccharide is glucosylated by ALG6, 8 and 10 before its transfer on the proteins. Deglycosylation by GCSI and GCSII during the quality control gives rise to a non-canonical Man_5_GlcNAc_2_ *N*-linked to proteins that structurally differs from the canonical Man_5_GlcNAc_2_ found in plants (Vanier et al., 2017) (**Figure 4**). A non-canonical Man_5_GlcNAc_2_ was also recently identified on proteins of *Scenedesmus* strains (Mócsai et al., 2025) and was also proposed for the main oligomannoside isolated from proteins of *Dunaliella salina* (Dehghani et al., 2024). This is likely a common feature of *chlorophyceae* of the green microalgae phylum (chlorophyte) that all exhibit a *C. reinhardtii*-like ALG repertoire (**Figure 3**).

For microalgae belonging to the other phyla, it appears, on the basis of the gene prediction, that the biosynthesis of the ER precursor occurs in *chlorellales* and *trebouxiophyceae* of the chlorophyte phylum and in *Euglena gracilis*, cryptophytes, haptophytes and rhodophytes as reported in plants and likely results in the completion of the biosynthesis of a Man_9_GlcNAc_2_ in the ER (**Figure 3**). However, biochemical characterization of their lipid-linked oligosaccharide precursors is still required to confirm these predictions.

Genes encoding OST subunits are also predicted in microalgae genomes but the inventory of subunits required for efficient transfer of the *N*-glycan precursor on proteins was not investigated intensively (Baïet et al., 2011; Mathieu-Rivet et al., 2013; Levy-Ontman et al., 2014; Liu et al., 2021; Behnke et al., 2021; Dehghani et al., 2024). Moreover, with regards to the *N*-glycosylation consensus sites, the analysis of glycopeptide sequences by mass spectrometry confirmed that glycans are *N*-linked to the asparagine residue of Asn-X-Ser/Thr *N*-glycosylation consensus sites as for other eukaryotes (Mathieu-Rivet et al., 2013; Vanier et al., 2015; Schulze et al., 2017, 2018; Oltmanns et al., 2020; Chen et al., 2022; Leprovost et al., 2024; Hammel et al., 2024).

### 3.2 Processing of *N*-linked glycans in the Golgi apparatus

#### 3.2.1 Trimming of mannose residues in the Golgi apparatus

**Figure 5:**
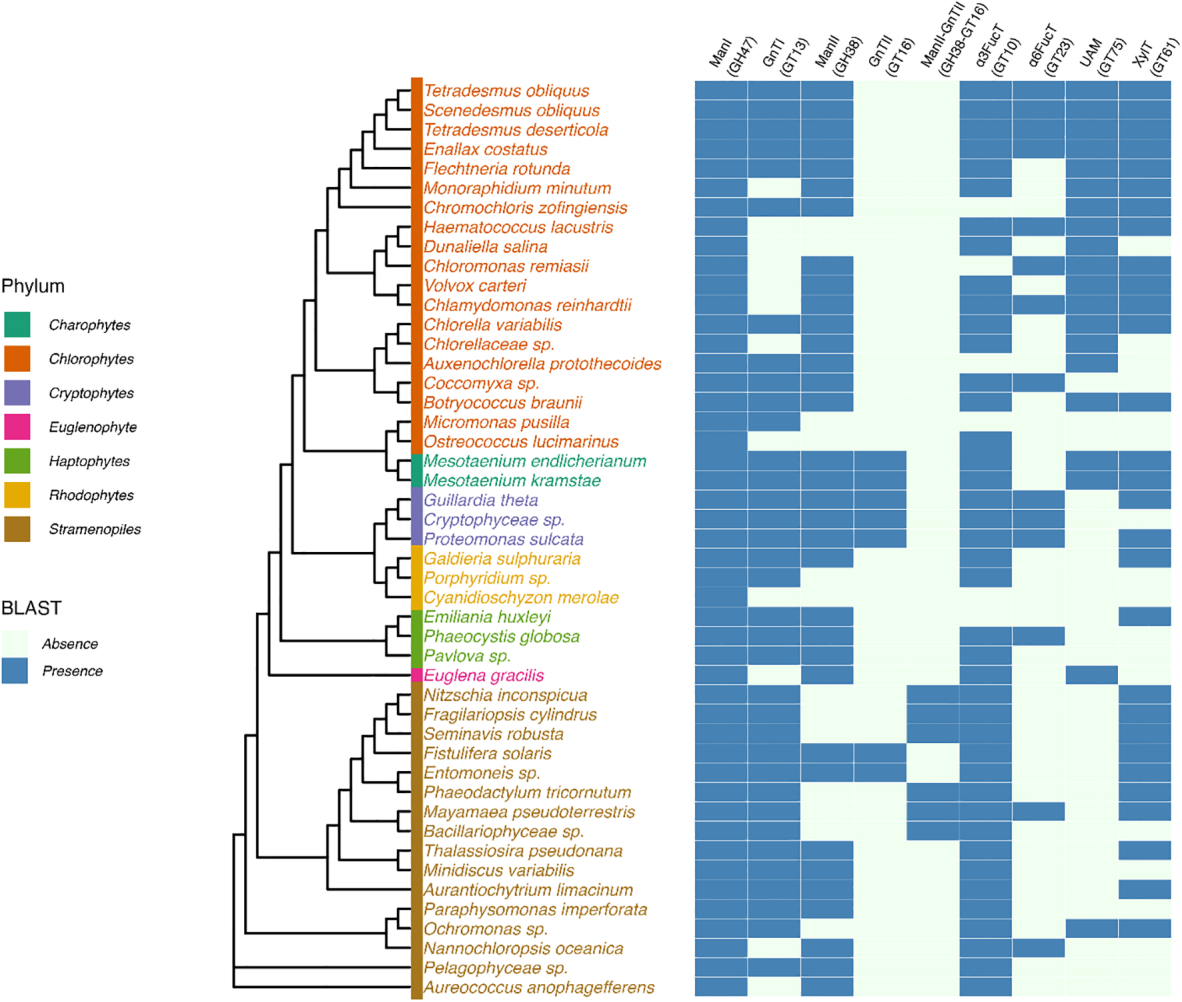
Prediction of putative Golgi enzymes of the *N-*glycosylation pathway in genomes of microalgae. Protein sequences were identified by BLASTp search in microalgae genomes using genes encoding Arabidopsis and human glyco-enzymes as query sequences. UAM, UDP-arabinopyranose mutase. UniProt assession numbers of proteins used as query sequence: *Arabidopsis thaliana*: Man I, D7R518; Man II, Q940T6; GnT I, F4JTL6; GnT II, Q9FT88; α3FucT, Q9LJK1; β2XylT, Q9LDH0; UAM, Q9SRT9; HEXO, Q8LFK0. *Homo sapiens*: α6FucT, Q9BYC5. See **Figure 3** for methodology used for the drawing of the cladogram.

In plants, properly folded glycoproteins continue their transit to the Golgi apparatus where the processing of oligomannoside Man_9_GlcNAc_2_ is initiated by the trimming of Man residues by mannosidase I isoforms (Man I, CAZy GH47). Whatever the order of outer Man removal, this trimming gives rise to a unique canonical Man_5_GlcNAc_2_ (**Figure 4**). With regards to microalgae, Man I isoforms are predicted in all microalgae genomes (**Figure 5**). In line with these predictions, different oligomannosides ranging from Man_3_GlcNAc_2_ to Man_8_GlcNAc_2_ were identified *N-*linked to microalgae proteins suggesting that the trimming of Man residues by Man I also occurs in these marine unicellular eukaryotes (Levy-Ontman et al., 2011; Baïet et al., 2011; O’Neill et al., 2017; Mócsai et al., 2019, 2025; Xie et al., 2021; Chen et al., 2022)). Moreover, in the diatom *P. tricornutum*, oligomannosides ranging from Man_5_GlcNAc_2_ to Man_9_GlcNAc_2_ were demonstrated to share identical structures to those of mammals and plants, thus suggesting that the mannose trimming of Man_9_GlcNAc_2_ by Man I occurs in a similar way in this diatom (Dumontier et al., 2021). However, the trimming of Man_9_GlcNAc_2_ by mannosidases may also provide some additional oligomannosides not found in plants as shown in *Chlorella* (Mócsai et al., 2019).

As noticed above, it should be pointed out that Man_5_GlcNAc_2_ identified in *Chlamydomonas reinhardtii*, *Dunaliella salina* and *Scenedesmus* strains (Vanier et al., 2017; Dehghani et al., 2024; Mócsai et al., 2025) exhibits a non-canonical structure having a linear trimannosyl sequence linked to the α(1,3)-mannose arm that is structurally different from the canonical Man_5_GlcNAc_2_ found in plants (**Figure 4**). This non-canonical oligomannoside derives from the removal of three Glc residues from the truncated Glc_3_Man_5_GlcNAc_2_ ER precursor (Vanier et al., 2017; Dehghani et al., 2024), whereas the canonical Man_5_GlcNAc_2_ *N-*linked to proteins in plants and *P. tricornutum* proteins results from the trimming of four Man residues from the Man_9_GlcNAc_2_ originating from the ER processing (Baïet et al., 2011) (**Figure 2**).

#### 3.2.2 Involvement of *N*-acetylglucosaminyltransferases in microalgae *N*-glycosylation pathways

In plants, after trimming of Man residues, the processing of *N*-linked glycans involved rebuilding steps catalyzed by *N*-acetylglucosaminyltransferases. The canonical Man_5_GlcNAc_2_ is stepwise processed by *N*-acetylglucosaminyltransferase I (GnT I, CAZy GT13), α-mannosidase II (Man II, CAZy GH38) and *N*-acetylglucosaminyltransferase II (GnT II, CAZy GT16) to yield the core GlcNAc_2_Man_3_GlcNAc_2_ (Strasser, 2016) (**Figure 4**). GnT I is a key GT in plants controlling the access to mature *N-*glycans. Plant Man II, GnT II and other Golgi GTs, such as the core α(1,3)-fucosyltransferase (core α3FucT) and core β(1,2)-xylosyltransferase (core β2XylT) involved in the Golgi *N-*glycan maturation, require glycan substrates carrying the terminal GlcNAc residue introduced by GnT I (**Figure 4**). Therefore, GnT I operates as a “stop-and-go” transferase and its inactivation results in the accumulation of Man_5_GlcNAc_2_ on plant proteins (von Schaewen et al., 1993). Search for gene encoding putative GnT I enabled the identification of candidate GT in many microalgae genomes suggesting the occurrence of plant-like GnT I-depending pathways in these unicellular organism (Baïet et al., 2011; Schulze et al., 2017) (**Figure 5**). The biochemical identification of GlcNAc-terminated oligomannosides in some *N*-glycan profiles of proteins from microalgae as reported in *Botryococcus braunii* (Schulze et al., 2017), *Chlorella vulgaris* (Mócsai et al., 2020a) and *P. tricornutum* (Xie et al., 2021) further supports the involvement of a GnT I in the biosynthesis pathway in these microalgae. However, it should be noted that terminal GlcNAc residue arising from GnT I action might be eliminated post Golgi maturation from *N-*glycans by hexosaminidases (HEXO, CAZy GH20). Thereby, GlcNAc-terminated *N*-glycans might only be transient intermediates in the glycosylation pathways of microalgae. This degradation of mature *N*-glycans by HEXOs was demonstrated in plants, insects and nematodes and yields Man_3_GlcNAc_2_-based oligosaccharides lacking the two terminal GlcNAc residues (Altmann et al., 1995; Gutternigg et al., 2007). By similarity with these processing and as illustrated in **Figure 4**, it was proposed that GnT I catalyses the transfer of a GlcNAc residue on a canonical Man_5_GlcNAc_2_ in *P. tricornutum*. Then, this terminal GlcNAc residue is likely removed by *N*-acetylglucosaminidases predicted in the *P. tricornutum* genome but biochemical experiments confirming this hypothesis are missing (**Figure 5**). *HEXOs* are also predicted in many microalgae genomes, together with a *GnT I* candidate, suggesting that this is a common feature in some microalgae as reported in plants and insect cells (**Figure 5**). As a consequence, on the basis of a protein *N-*glycan profile, it is highly speculative to conclude about the occurrence of a GnT I-dependent pathway in a given microalgae because this transient terminal GlcNAc residue on the α(1,3)-Man that is diagnostic for a Golgi GnT I activity, might have been eliminated post-Golgi maturation by HEXOs.

After transfer of a terminal GlcNAc by GnT I, further Golgi processing of *N-*glycans in plants involves Man II and GnT II that successively remove two Man residues on the α(1,6)-Man and then transfer a second terminal GlcNAc unit to give rise to a core GlcNAc_2_Man_3_GlcNAc_2_ (**Figure 4**). In diatoms, genes encoding Man II and GnT II are also predicted in genomes (**Figure 5**). Surprisingly, these two sequences are integrated in a single bifunctional enzyme in *P. tricornutum* (**Supplementary Figure 1**). In this diatom, data collected from RNA-sequencing analyses suggested that this bifunctional sequence is expressed as a single transcript (Ovide et al., 2018). The bifunctional *P. tricornutum* Man II/GnT II is a type II membrane protein of 1453 amino-acids with a predicted Man II domain located on the *N*-terminal end which exhibits 49% of identity with Man II from *Drosophila melanogaster* (van den Elsen et al., 2001). GnT II catalytic domain is located on the C-terminal end of the predicted protein in *P. tricornutum* and exhibits the catalytic amino-acids of human GnT II (Kadirvelraj et al., 2018) (**Supplementary Figure 1**). Such a bifunctional glyco-enzyme is likely rare in microalgae while the fusion of 3,5-epimerase (RmlC) and 4-reductase (RmLD), two enzymes of the NDP-L-rhamnose biosynthesis pathway, was described in the haptophyte *Prymnesium parvum* (Wagstaff et al., 2019).

This unusual Golgi *N*-glycan processing seems to also occur in other diatoms as genes encoding homologous bifunctional enzymes are predicted in genomes of *Fragilariopsis cylindrus*, *Nitzschia inconspicua*, *Seminavis robusta*, *Bacillariophyceae* sp. and *Mayamaea pseudoterrestris* with conserved motifs for both Man II and GnT II activities (**Figure 5**). While biochemical data are still missing, we postulate that in diatoms after transfer of a first GlcNAc by GnT I on the canonical Man_5_GlcNAc_2_, bifunctional Man II/GnT II enables the biosynthesis of a core *N*-glycan carrying two terminal GlcNAc residues as demonstrated in plants. The two terminal GlcNAc arising from GnT I and II activities are then likely eliminated by HEXOs (Baïet et al., 2011) (**Figure 4**).

In contrast to *P. tricornutum*, it was established that the *N*-glycosylation of proteins in *C. reinhardtii* and *D. salina* occurs in a GnT I-independent pathway (Vanier et al., 2017; Dehghani et al., 2024). Indeed, *C. reinhardtii* and *D. salina* lack Golgi GnT I and synthesize a non-canonical Man_5_GlcNAc_2_ arising from their ER truncated lipid-linked oligosaccharide that is not substrate for GnT I (Vanier et al., 2017) (**Figure 4**). For other chlorophytes exhibiting the same ALG repertoire, additional biochemical investigations are needed to confirm that they also *N*-glycosylate their proteins through a GnT I-independent pathway. For instance, a *GnT I* gene (and *HEXO*) is predicted in the *Scenedesmus* genome despite the prediction of ALG3, 9 and 12 in this microalga and the presence of non-canonical Man_5_GlcNAc_2_ on its proteins (Mócsai et al., 2025). Same *GnT I* and *ALG* repertoires are also predicted in *Chromochloris, Enallax, Flechtneria* and *Tetradesmus*.

#### 3.2.3 Maturation of *N*-glycans in the Golgi apparatus

Independently of the involvement of a GnT I, final steps of the processing of *N-*linked glycans in microalgae involve the transfer of Fuc, Xyl, hexose and/or arabinose (Ara) residues on oligomannosides (**Figure 4**). In *Volvox carteri*, Man_3_GlcNAc_2_ is substituted by a core Xyl (Balshüsemann and Jaenicke, 1990). Man_3_GlcNAc_2_ *N-*glycan carrying an α(1,3)-fucose residue on the proximal GlcNAc was identified on proteins isolated from *P. tricornutum* (Baïet et al., 2011; Zhang et al., 2019). In *C. reinhardtii*, non-canonical Man_5_GlcNAc_2_ is decorated by a core α(1,3)-fucose and two Xyl residues, one being a core β(1,2)-linked xylose (Mathieu-Rivet et al., 2013; Schulze et al., 2018; Lucas et al., 2020; Oltmanns et al., 2020). Proteins from *Porphyridium* sp. are *N-*glycosylated with oligomannosides substituted with one or two Xyl residues, the first one being attached to the penultimate GlcNAc residue of the chitobiose motif (Levy-Ontman et al., 2011) (**Figure 1**). Dixylosylated *N-*glycans were also reported in *Scenedesmus* with a first Xyl residue located on the penultimate GlcNAc and the second one to the β-mannose of the core (Mócsai et al., 2021) (**Figure 1**). In addition, Man_3_GlcNAc_2_ *N-*glycan carrying a core α(1,6)-fucose was recently described in *Scenedesmus* strains among other mature *N*-glycans, some of them exhibiting a core α(1,3)-fucose. This is an unexpected discovery because core α(1,6)-fucosylation of *N*-glycans is only known to date in animal glycoproteins (Mócsai et al., 2025). The in-depth biochemical analysis of protein *N*-linked glycans from *Chlorella* species were performed by liquid chromatography on porous graphitic carbon column coupled to mass spectrometry (LC-MS) and nuclear magnetic resonance (NMR) spectroscopy. These investigations revealed *O-*methylated oligomannosides, as well as an unsuspected variety of new *N*-glycans composed of short oligomannosides substituted by Gal*f* and Gal*p*, as well as Ara*f* and Ara*p* units (Mócsai et al., 2020a, 2020b, 2021, 2024) (**Figure 1**).

#### 3.2.4 *O*-methylation and *O*-aminoethylphosphonylation

Several glycans *N*-linked to proteins of microalgae are *O-*methylated on Man residues (Levy-Ontman et al., 2011; Mathieu-Rivet et al., 2013; Schulze et al., 2018; Oltmanns et al., 2020; Lucas et al., 2020; Mócsai et al., 2019, 2024, 2025). Di *O*-methylation of Man was also observed in some *N*-glycans (Schulze et al., 2017; Mócsai et al., 2019). *O-*methylation is not restricted to Man but also occurs on the Fuc residue linked to the proximal GlcNAc residue as shown for the recombinant human erythropoietin expressed in *C. reinhardtii* (Leprovost et al., 2024). *O-*methylation of glycans was previously described in worms and mollusks, and more frequently in bacteria and fungi (Hykollari et al., 2022). Such *O-*methylations are absent in plant glycoproteins and their biological significance is still unknown. However, we assume that in a biotechnological context they may be immunogenic in humans as shown for other *O-*methylated sugars of glycoconjugates or polysaccharides (Siddiqui et al., 2007; De Ruiter et al., 1994). In *Euglena gracilis*, instead of *O-*methylation of Man, *O*-aminoethylphosphonylation occurs on Man_8_GlcNAc_2_ and Man_9_GlcNAc_2_ *N-*linked glycans (O’Neill et al., 2017) (**Figure 1**). Such an unusual substitution of *N-*glycans was previously reported in insect glycoproteins (Hard et al., 1993).

### 3.3 Biochemical characterization and Golgi localization of glycosyltransferases

It is widely accepted that the processing of glycans *N*-linked to proteins takes place in the Golgi apparatus through the stepwise action of Golgi-resident GHs and GTs that exhibit distinct substrate specificities. In plants, the *N*-glycosylation pathway is controlled by a subtle sub-compartmentalization of enzymes in Golgi stacks, thereby maintaining consecutive and selective processing steps (Schoberer and Strasser, 2011). In contrast, the substrate specificity and cellular localization of glyco-enzymes involved in the protein *N-*glycosylation pathways in microalgae received little attention to date. GnT I and a GDP-fucose transporter from *P. tricornutum* were demonstrated to be able to complement CHO glycosylation mutants (Baïet et al., 2011; Zhang et al., 2019). Lec 1 CHO mutant lacks GnT I activity and accumulates Man_5_GlcNAc_2_ *N*-linked to its proteins. The efficient complementation by *P. tricornutum* GnT I of Lec 1 CHO mutant indicated that the diatom transferase is properly targeted to the Golgi apparatus of CHO cells where it is able to use a canonical Man_5_GlcNAc_2_ as substrate (Baïet et al., 2011). As well, the expression of the *P. tricornutum* GDP-fucose transporter was able to rescue the fucosylation of proteins in the gmt5 CHO mutant lacking its endogenous Golgi transporter (Zhang et al., 2019).

Search for gene encoding Golgi-resident enzymes involved in the transfer of biochemically characterized core *N*-glycan decorations allowed the identification of putative core α3FucT (CAZy GT10) and core β2XylT (CAZy GT61) in microalgae genomes (Baïet et al., 2011; Schulze et al., 2017; Mathieu-Rivet et al., 2013; Behnke et al., 2021; Liu et al., 2021). Investigation of 40 additional genomes confirms that these two GTs are widely distributed in microalgae, except for rhodophytes despite the identification of xylosylated *N*-glycans in *Porphyridium* sp. (**Figures 1** and **5**), suggesting the existence of XylTs in other families than GT61.

As green microalgae are considered to be the ancestor of land plants, core α(1,3)-fucosylation and core β(1,2)-xylosylation of their protein *N*-glycans is not unexpected. By contrast, the recent identification of core α(1,6)-fucosylated *N*-glycans in *Scenedesmus* strains is more surprising as this glyco-epitope is to date only known in animals (**Figure 1**) (Mócsai et al., 2025). This unexpected core decoration is likely not restricted to *Scenedesmus* because genes encoding core α(1,6)-FucT (core α6FucT, CAZy GT23) are also predicted in genomes of different microalgae phyla (Mócsai et al., 2025) (**Figure 5**). Other genes encoding putative GTs have also been predicted in microalgae genomes (Liu et al., 2021). However, these predictions are not supported by any analytical experiments performed on *N-*glycans. Biochemical evidences for the involvement of predicted FucT and XylT in core α(1,3)-fucosylation and core β(1,2)-xylosylation of protein *N-*glycans were reported to date in *C. reinhardtii* taking advantage of the availability of glycosylation mutants. *C. reinhardtii* mutants impaired in FucT and XylT exhibited drastic decreases of *N*-glycan core α(1,3)-fucosylation and xylosylation of their total proteins (Schulze et al., 2018; Lucas et al., 2020; Oltmanns et al., 2020). Moreover, the involvement of the putative core α3FucT from *P. tricornutum* in the core fucosylation was confirmed by overexpression of this Golgi transferase in this diatom and observation of a large increase of the immunodetection by western-blot of core α(1,3)-fucose epitopes on proteins of the transformants (Zhang et al., 2019).

Whilst bio-informatics investigation and biochemical data allow to depict a first picture of the microalgae *N*-glycosylation pathways, many pieces of the puzzle are still missing. It is therefore speculative to draw plausible glycosylation pathways even for microalgae for which the structure of lipid-linked oligosaccharide and mature *N-*glycans were described (**Figure 4**). For instance, in GnT I-dependent pathways, the substrate specificities of core α3FucT, core α6FucT and core β2XylT are unknown, notably their requirement for GlcNAc-terminated substrates resulting from GnT I activity as reported in plants. In GnT I-independent processing, the structure of oligomannoside substrates for XylT and FucT is also questionable. It is to note that we cannot exclude that GnT I-dependent or independent pathways may coexist. For instance, xylosylation of *N-*glycans occur on Man_9_GlcNAc_2_ *N*-linked to *Porphyridium* proteins despite the prediction a *GnT I* in its genome (Levy-Ontman et al., 2011; Liu et al., 2021).

Information on the cellular localization of candidate Golgi GTs in microalgae phyla is also very weak to date. Glycoproteins carrying core β(1,2)-xylose epitopes were immunodetected in the cis-Golgi compartment of the *C. reinhardtii* Golgi apparatus, suggesting that core β(1,2)-xylosylation of proteins of this green microalgae occurs in this cellular compartment or even before in the ER (Ropitaux et al., 2024). Moreover, GnT I and core α3FucT from *P. tricornutum* were localized in the Golgi apparatus by imaging by transmission electron microscopy of diatoms expressing tagged GTs (Zhang et al., 2019). The putative core β2XylT from *P. tricornutum* was localized by confocal microscopy in spots that were proposed to be Golgi apparatus but immunolocalization by transmission electron microscopy needs to be performed for confirmation (Liu et al., 2016). Note that GnT I, core α3FucT and core β2XylT are type II proteins exhibiting a transmembrane domain in the N-terminal end allowing their membrane anchoring as for plant homologues (Schoberer and Strasser, 2011). Two other *P. tricornutum* FucTs were proposed to be located in the plastid stroma but this cellular location is questionable to date (Xie et al., 2023).

### 3.4 *N*-glycan pentosylation in plant and microalgae

One of the noteworthy structural features of plant and microalgae *N*-glycosylation pathways is the decoration of mature *N*-glycans with Xyl or Ara, two pentose residues. Before their transfer by Golgi transferases to specific substrates, activated nucleotide pentoses are first obtained *via* the nucleotide sugar interconversion pathway. In plants, D-Xyl is the unique pentose found on *N*-glycans. UDP-α-D-xylopyranose (UDP-α-D-Xyl*p*) is produced in plants by decarboxylation of UDP-α-D-glucuronic acid (UDP-α-D-GlcA). The biosynthesis of UDP-β-L-arabinopyranose (UDP-β-L-Ara*p*) is then achieved from UDP-α-D-Xyl*p* by action of an UDP-Xyl 4-epimerase, a reaction that takes place in the plant Golgi lumen (Figueroa et al., 2021). In plant glycoproteins, a xylopyranose residue is then transferred from UDP-α-D-Xyl*p* to the β-mannose of the core *N*-glycan by a core β2XylT (**Figure 1**). It should be noted that Priem and coll. described in 1993, among other free oligosaccharides isolated from tomato, a novel *N*-glycan in which the core β(1,2)-xylopyranose is replaced by a core α(1,2)-arabinofuranose residue (Priem et al., 1993). However, arabinose-containing *N*-glycans in plants has never been reported on other plant proteins. We may assume that the presence of isobaric core α(1,2)-arabinofuranose in plant *N*-linked glycans may have escape to plant *N*-glycome analyses as plant *N*-glycan profiling is usually performed by mass spectrometry. Nevertheless, without any new data on these arabinose-containing oligosaccharides, the core α(1,2)-arabinofuranosylation of plant *N*-glycans remains a matter of debate. With regards to these putative arabinosylated *N*-glycans, as well as other arabinofuranose-containing glyco-molecules, it should be noted that the involvement of a cytosolic UDP-L-arabinopyranose mutase (UAM, CAZy GT75) and a UDP-β-L-Ara*f* transporter are required to supply plant Golgi arabinofuranosyltransferases (Ara*f*Ts) with the appropriate nucleotide-sugar UDP-β-L-Ara*f* (Rautengarten et al., 2017; Saqib et al., 2019). Indeed, UAM is able to convert UDP-β-L-Ara*p* into its isomer UDP-β-L-Ara*f*. Note that UAM belongs to the Reversibly Glycosylated Polypeptides (RGPs) gene family (Kuttiyatveetil and Sanders, 2017) and this reversible self-glycosylation activity led to their classification in the CAZy GT75 family.

In microalgae, pentosylation of protein *N*-glycans is not restricted to the core β(1,2)-xylosylation as in plants but is more extensive with β-xylopyranose, α/β-arabinofuranose and α/β-arabinopyranose units linked to the Man or GlcNAc residues of the core or to outer Man as illustrated in **Figure 1**. With regards to the enzyme machinery required for the biosynthesis of such pentosylated *N*-glycans, UDP-GlcA decarboxylase and UDP-Xyl 4-epimerase are predicted in most microalgae (Gügi et al., 2015) but biochemical data are missing to confirm their role, as well as their cellular localization. Investigation of microalgae genomes, using Arabidopsis UAM as a query sequence, enabled the prediction of genes encoding UAM in streptophytes and *Euglena gracilis*, as well as in chlorophytes including *Chlorella* species in which Ara*f*-containing *N*-glycans were biochemically characterized (**Figure 5**). These UAM show high homologies with plant mutases (Fedosejevs et al., 2017). Moreover, one UAM was biochemically characterized in *C. reinhardtii* (Kotani et al., 2013). This enzyme was proposed to supply the Golgi apparatus in UDP-β-L-Ara*f* for the biosynthesis of α(1,2)- and α(1,3)-L-arabinofuranose glycan motifs of hydroxyprolin-rich glycoproteins in this green microalgae.

XylTs are predicted in most microalgae genomes (**Figure 5**) although information on their involvement in *N*-glycan biosynthesis is missing in some microalgae as for instance in *P. tricornutum*. Microalgae XylTs belong to the CAZy GT61 family as for plant XylTs. However, the function of putative XylTs as *N*-glycan-specific GTs was only confirmed in *Chlamydomonas* (Lucas et al., 2020; Oltmanns et al., 2020; Leprovost et al., 2024). It should be noted that the GT61 family gathers both xylopyranosyltransferases and arabinofuranosyltransferases which suggests that these transferases may use either UDP-α-D-Xyl*p* or UDP-β-L-arabinofuranose (UDP-β-L-Ara*f*), two structurally related nucleotide sugars. This hypothesis is supported by the identification of β(1,2)-xylopyranose- or α(1,2)-arabinofuranose residues in a plant *N*-glycan population suggesting that plant XylTs may catalyze the biosynthesis of the two isobaric oligosaccharides depending on the availability of the corresponding nucleotide (Priem et al., 1993). For microalgae *N*-glycans containing either a β-arabinofuranose or a α/β-arabinopyranose residue, investigation of specific arabinosyltransferases has to be performed to unravel the biosynthesis of these particular glycan motifs. As illustrated in **Figure 1**, there is a notable diversity in the linkage type of the pentoses residues to the *N*-glycans suggesting the involvement of GTs families beyond the GT61.

## 4 Conclusion

A wide variety of mature glycans structures have been characterized already in microalgae whilst the investigation of glycans *N*-linked to their proteins was only initiated recently. Many other mature *N*-linked oligosaccharides carrying various pentose(s) and deoxyhexose(s) were also detected but not structurally characterized (Mócsai et al., 2025; personal communication of the authors). This indicates that the microalgae *N*-glycan diversity is far from being revealed. To date, non-canonical Man_5_GlcNAc_2_, core α(1,3)- or core α(1,6)-fucosylation, multiple pentosylations and *O*-methylations are the most noteworthy structural features of protein *N*-glycans. Bioinformatics investigation of genomes suggested that microalgae within a phylum share a common set of glyco-enzymes enabling their classification in a *N*-glycan sub-family having one or more of these specific glycan motifs (**Figures 3** and **5**). It should be emphasized that, considering the wide diversity of closely related oligosaccharide sequences, the investigation of protein *N*-glycans from microalgae will need the implementation of appropriate analytical methodologies for in depth profiling of proteins from other microalgae, as well as the re-evaluation of glycan profiles of already investigated strains. Notably, efficient chromatography methodologies, as well as ion mobility mass spectrometry, have to be performed for the separation of isobaric *N*-glycans, in complement to the glycan sequencing by MS^2^ fragmentation or structural analysis by NMR spectroscopy.

Although microalgae and plant *N*-glycans share some common glyco-epitopes, such as core α(1,3)-fucose and core β(1,2)-xylose, microalgae *N*-glycans largely diverge from those of plants and this likely results from their divergent evolution. It is commonly accepted that first functions performed by the glycosylation of proteins is the increasing of its hydrophily and it protective effect against proteases by masking proteolytic sites. However, the diversity of glycan decorations on microalgae proteins suggests that, beyond these essential physicochemical functions, these glycans might exert more specific biological roles. As proposed by Mócsai et al. (2021), specific glycans *N*-linked to proteins located at the microalgae cell surface might be responsible for the specific interaction with mating partners to assure sexual reproduction or with symbiotic bacteria that are essential for microalgae growth. Considering this wide diversity of protein *N*-glycan profiles, F. Altmann and co-workers suggested to use it as trait for taxonomic classifications (Mócsai et al., 2020a, 2020b, 2024).

To unravel the *N*-glycosylation pathways in microalgae, many efforts have also to be made to identify GTs, their cellular localization, as well as their substrate specificity. This could be achieved through the implementation of bioassays on various substrates performed with recombinant GTs lacking their transmembrane domain expressed in insect, plant or mammalian cells. The complementation of plant or mammalian cell *N*-glycosylation mutants should also provide major information on microalgae GTs. Among main items of concern, the substrate specificity of GnT I from these unicellular organisms has to be investigated because this transferase plays a pivotal role in protein *N*-glycosylation pathways. Moreover, understanding the arabinosylation and/or xylosylation process of *N*-glycans in microalgae will also be a major challenge because it concerns the transfer of α/β-arabinofuranose and α/β-arabinopyranose at various locations that require both the synthesis of UDP-β-L-Ara*p* and UDP-β-L-Ara*f*, and their transfer by Golgi GTs that are likely not restricted to members of the GT61 family.

In a biotech context aiming at producing microalgae-derived biologics having human-compatible glycans, the glycoengineering of microalgae *N*-glycosylation pathways will likely be a tricky task. If human mature *N*-glycans are not required for its biological activity, the retention of the biologics in the ER by fusion to an ER retention signal may enable the protein to be *N*-glycosylated by oligomannosides and prevent the protein to acquire immunogenic mature *N*-glycans as recently demonstrated for the hepatitis C virus glycoprotein produced in *Porphyridium* (Hammel et al., 2024). Getting human-compatible mature glycans *N*-linked to biologics expressed in microalgae will require the inactivation of several Golgi GTs through gene editing methodologies or the selection of *N*-glycosylation mutants from libraries, assuming that the target enzymes were previously well-characterized. To date, inactivation of core α(1,3)-fucosylation and core β(1,2)-xylosylation of protein *N-*glycans were successfully performed in *C. reinhardtii* taking advantage of the availability of glycosylation mutants (Schulze et al., 2018; Lucas et al., 2020; Oltmanns et al., 2020; Leprovost et al., 2024). These mutant lines do not exhibit detectable growth phenotypes suggesting that the alteration of *N*-glycan maturation steps do not affect the cell viability and thus, the selection of glycosylation mutants may be considered as a plausible solution. When multiple xylosylations, arabinosylations or fucosylations occur as illustrated in **Figure 1**, an alternative strategy to the inactivation of Golgi-resident transferases would be to target the biosynthesis of the nucleotide-sugar in the cytosol. After import in the Golgi apparatus by specific transporters, nucleotide-sugars are used by GTs to perform the glycosylation of proteins in the secretory system. Thus, their deletion by inactivation of a key step of their biosynthesis will therefore impair GT activity. This strategy was not considered as a pertinent glycoengineering strategy in plants to erase core α(1,3)-fucosylation and core β(1,2)-xylosylation on glycoproteins, the two main immunogenic glycoepitopes. Indeed, impairment of the biosynthesis of the corresponding nucleotide-sugars, GDP-Fuc and UDP-Xyl, induce drastic growth defects because these monosaccharides are crucial for the cell wall xylan, xyloglucan and rhamnogalacturonan II integrity. So far, little is known about the structure of Fuc-, Ara- and Xyl-containing glyco-polymers in microalgae and to what extend the impairment of their biosynthesis may induce growth defects or microalgae lethality.

With regards to the implementation of knock-in strategies, complementation of microalgae with heterologous GHs or GTs was not reported so far. However, the efficient complementation of CHO glycosylation mutants with *P. tricornutum* GnT I and GDP-fucose transporter demonstrated that targeting of Golgi enzymes is well-conserved between microalgae and mammalian cells (Baïet et al., 2011; Zhang et al., 2019). This suggests that complementation of microalgae with plant or mammalian enzymes would also be successful. However, a better understanding of *N*-glycan processing and the localization of endogenous Golgi-resident GTs is needed to properly address the recombinant GT.
