# Supplementary material for: An overview of protein N-glycosylation diversity in microalgae
Source: Front Plant Sci. 2025 Sep 25;16:1669918. doi: 10.3389/fpls.2025.1669918 (PMC12507850; doi:10.3389/fpls.2025.1669918)
Supplement: Supplementary file 1 [file Table1.docx]

Supplementary Material

**Supplementary Figure 1.** A) schematic representation of PtMan II/GnT II (Phatr3_52248). Sequence alignments of Man II/GnT II from *P. tricornutum* with (B) *Drosophila melanogaster* Man II (van den Elsen *et al*., 2001) and (C) human GnT II (Kadirvelraj et al., 2018). Conserved motifs and catalytic amino-acids are indicated. In the Man II domain, a zinc ion is involved in a pentavalent structure with H_90_, D_92_, D_204_, D_472_ and H_471_, aspartic acid D_204_ being responsible for the hydrolysis of the mannosyl residues (van den Elsen *et al*., 2001). In the GnT II domain, E_259_ED_261_ motif and H_374_ are involved in the interaction with phosphate residues of UDP-GlcNAc *via* a Mn^2+^ ion and D_342_DYN_348_ motif as well as W_346_ and E_259_ involved in the recognition of the *N*-glycan substrate (Fig. S6c) (Kadirvelraj et al., 2018).
